# How do teacher support trajectories influence primary and lower-secondary school students’ study well-being

**DOI:** 10.3389/fpsyg.2023.1142469

**Published:** 2023-08-24

**Authors:** Sanna Ulmanen, Pihla Rautanen, Tiina Soini, Janne Pietarinen, Kirsi Pyhältö

**Affiliations:** ^1^Faculty of Education and Culture, Tampere University, Tampere, Finland; ^2^Centre for University Teaching and Learning, Faculty of Educational Sciences, University of Helsinki, Helsinki, Finland; ^3^School of Applied Educational Sciences and Teacher Education, Philosophical Faculty, University of Eastern Finland, Joensuu, Finland; ^4^Department of Curriculum Studies, University of Stellenbosch, Stellenbosch, South Africa

**Keywords:** teacher support trajectories, study engagement, study-related burnout, grade-level differences, latent growth mixture modeling

## Abstract

Effective long term teacher support is key to promoting and sustaining students’ study well-being at school. However, little is known about individual variations in the development of perceived teacher support and how such variations are associated with study engagement and study-related burnout. Also, understanding of the differences between age cohorts across school levels is still limited. To address this limitation, we used latent growth mixture (LGM) modeling to study whether teacher support trajectories influenced study engagement and study-related burnout among Finnish primary and lower-secondary school students. Two cohorts of students, namely primary school students from the 4th to 6th grades (*N* = 2,204) and lower-secondary school students from the 7th to 9th grades (*N* = 1,411), were followed for three years. LGM revealed four latent trajectories for teacher support, which were labeled *high stable* (72%), *low stable* (12%), *decreasing* (11%) and *increasing* (5%). The teacher support trajectories were strongly associated with students’ study engagement and study burnout. Moreover, heightened study-related burnout symptoms and decreased study engagement were associated with a decline in perceived teacher support, while higher levels of study engagement and low levels of study burnout symptoms were associated with a continuum of positive teacher support experience. Primary school students were more likely to employ stable and high levels of teacher support, compared with lower-secondary school students, highlighting the importance of improving conditions in lower-secondary school so that the teacher support will better reach all their students.

## 1. Introduction

Teachers play a crucial role in promoting students’ adjustment to school, as well as to society later in life (Reschly et al., 2008; You and Sharkey, 2009; Lewis et al., 2011). Teacher support has been shown to promote school achievement, study engagement and decrease risk for developing study burnout (Wang and Eccles, 2012; Estell and Perdue, 2013; Liu et al., 2016; Quin, 2017; Virtanen et al., 2018; Weyns et al., 2018). Teacher support has also shown to promote students’ engagement in prosocial behavior among their peers (Hughes and Chen, 2011; Luckner and Pianta, 2011; Ulmanen et al., 2016a). Despite the importance of teacher support throughout students’ school years, a significant variation has been found in students’ teacher support experiences. Also, the developmental trends of experienced teacher support have been shown to vary between students (O'Connor, 2010; Split et al., 2012; Bosman et al., 2018; Özdemir and Özdemir, 2020). However, understanding the variation in the developmental trajectories of teacher support experiences among different age cohorts across school levels is limited. Even less is known about the dynamics of the teacher support experience trajectories in relation to study well-being. To identify the students at risk and proactively prevent their disengagement from studies, better understanding of the support trajectories and their influence on students’ study well-being across the grades is an imperative. To tackle the challenge, we use latent growth mixture modeling to explore whether Finnish primary and lower-secondary school students’ teacher support trajectories influence their study well-being. The data were obtained by following for three years two cohorts of students, namely those in primary school from 4th to 6th grade (*N* = 2,204) and lower-secondary school from 7th to 9th grade (*N* = 1,411).

### 1.1. Teacher support and study well-being

Teacher support refers to the availability of social resources in teacher–student relationships that help students to cope with challenging school-related tasks or overcome negative events (Cohen and Syme, 1985; Cohen et al., 2000; Wentzel et al., 2016). In particular, emotional and informational support seem to be important factors in coping with study-related issues (Wang and Eccles, 2012; Liu et al., 2016; Wentzel et al., 2016; Ulmanen et al., 2016b; Havik and Westergård, 2019). Emotional support refers to the care, acceptance, acknowledgement and encouragement received from teachers, while informational support refers to the constructive feedback, advice and affirmation that they provide (House, 1981; Malecki and Demaray, 2002; Ulmanen et al., 2016b).

Teacher support has been found to be one of the most significant sources of school-related support for students throughout the school years. It has been found to have both immediate and long-term effects on students’ academic, emotional and behavioral adjustment to school (e.g., Wang and Eccles, 2012; Estell and Perdue, 2013; Ciarrochi et al., 2017; Aldrup et al., 2018; Hughes and Cao, 2018; Weyns et al., 2018). For example, Roorda et al. (2011) found in their meta-analysis of 99 studies strong evidence that affective teacher–student relations promote students’ engagement and achievement in studies all the way through to older students in late adolescence. Similarly, Quin (2017) found in a systematic review that high-quality teacher–student relations contribute to students’ engagement, school achievement, school attendance and decreased risk for disruptive behaviors, suspension and dropout. In turn, Kim et al. (2018) found in their meta-analysis of 19 studies that teacher support has a strong negative relation to student burnout. We recently found through profile analysis that a lack of perceived teacher support is related to increased levels of study burnout symptoms and that support from other sources is not enough to compensate for lack of teacher support in this regard (Ulmanen et al., 2022a,b, 2023).

There is increasing research evidence that the interrelationship between teacher support and study well-being may be reciprocal (Currie, 2014; Pitzer and Skinner, 2017; Zee et al., 2021). It has been found that engaged students are more likely to perceive their teachers as supportive than do their less engaged peers (O'Connor, 2010; Ouweneel et al., 2011; Nurmi and Kiuru, 2015; Yang et al., 2021). In a recent study using a random intercept cross-lagged panel model, we found that study engagement seems to be a stronger, more consistent predictor for later teacher support than the reverse being true (Rautanen et al., 2022). However, the relationship between teacher support and study wellbeing is complex and not yet fully understood. Previous studies that have systematically explored both teacher support and study well-being longitudinally have mostly applied a variable-center approach. Little is known about how individual teacher support trajectories are related to students’ study well-being (throughout the whole study period) or whether study well-being at the beginning of a study period is associated with change in teacher support. The aim of this study is to examine the individual changes in students’ teacher support and their association with study well-being experiences over time.

In this study, study well-being is examined as a combination of positive study-related mental states, such as study engagement, and an absence of negative ones, such as study burnout. Study engagement refers to positive and fulfilling study-related experiences, characterized by a sense of energy, dedication, and absorption in studying (Salmela-Aro and Upadyaya, 2012; Upadyaya and Salmela-Aro, 2013). While energy refers to vigor and mental resilience while studying (e.g., Schaufeli et al., 2002; Salmela-Aro and Upadyaya, 2012), dedication refers to a sense of enthusiasm and identification regarding studies and also to perceiving schoolwork as meaningful (e.g., Tuominen-Soini and Salmela-Aro, 2014; Salmela-Aro, 2017). Absorption refers to feelings of competence and being fully and happily concentrated on studying (e.g., Schaufeli and Bakker, 2004; *cf.* the concept of flow in Csikszentmihalyi, 1990). In turn, study burnout syndrome has three distinctive but complementary symptoms, namely exhaustion, entailing a state of strain and chronic fatigue; cynicism, characterized by a loss of interest in schoolwork and not seeing school as meaningful; and a sense of inadequacy in studying, entailing a diminished sense of competence in terms of studying at school (Maslach et al., 2001; Schaufeli et al., 2002; Salmela-Aro et al., 2016). Study burnout develops gradually, meaning that a student may experience exhaustion due to exposure to high workload but not have a cynical attitude toward studying (Salmela-Aro and Upadyaya, 2014). Study engagement and study burnout are complementary but distinct aspects of students’ overall study well-being. High levels of study engagement and low levels of study burnout are thought to be the ideal combination for optimal study well-being. However, it is possible for students to experience high levels of study engagement while simultaneously feeling exhausted (e.g., Tuominen-Soini and Salmela-Aro, 2014; Ulmanen et al., 2022a,b, 2023). Despite there being numerous studies of the individual trajectories of students’ teacher support and of study well-being, only a few have looked at both of these simultaneously throughout the study period (e.g., Ulmanen et al., 2022a,b). In this study, both aspects of study well-being are considered.

### 1.2. Individual differences in the development of teacher support

Generally, students’ perceived teacher support has been found to decrease over time. Older students have been found to report lower support than younger ones (Malecki and Demaray, 2002; Bokhorst et al., 2010; De Wit et al., 2011; Havik and Westergård, 2019; Rautanen et al., 2022). However, studies applying a person-centered approach have found that not all students employ the same trajectory and there are differences in the developmental trends of teacher support between students (Özdemir and Özdemir, 2020; Ulmanen et al., 2022a, 2023; see also O'Connor, 2010; Split et al., 2012; Bosman et al., 2018). Typically, teacher support is perceived to be quite stable or slightly decreasing over time. Most students have been found to perceive teacher support as consistently high or moderate throughout their school years; that is, they receive constructive feedback and advice and are treated fairly and emotionally warmly by teachers (Split et al., 2012; Bosman et al., 2018; Özdemir and Özdemir, 2020; Ulmanen et al., 2022a). However, some continuously perceive teachers’ support as insufficient and inappropriate and feel that their ideas and opinions are not valued or respected by teachers (Split et al., 2012; Bosman et al., 2018; Özdemir and Özdemir, 2020). Besides stable trajectories, significant changes in perceived teacher support have also been identified, while some students’ experiences of teacher support fall or increase significantly during their school years (Ulmanen et al., 2022a, 2023).

Despite the numerous studies of the variation of supportiveness of teacher–student relationships, with few exceptions (e.g., Reddy et al., 2003; Longobardi et al., 2016; Özdemir and Özdemir, 2020; Ulmanen et al., 2022a,b), previous research has primarily focused on the early childhood or primary school period. There is a lack of understanding of whether there are differences in teacher support trajectories among students from different grade levels. Compared with younger students, older students go through stronger physical and emotional changes as they mature. They may also face increased pressure to perform academically at school. In addition, the structure of school organization differs between different grade levels. Younger students are typically taught by one teacher who is responsible for teaching a range of subjects, whereas older students are taught by several subject teachers during the day, which can make it more challenging for the teachers to provide individualized support and for the students to seek out support (see Jindal-Snape et al., 2021). To understand the importance of the individual and environmental changes for students’ teacher support trajectories, in this study, both primary and lower-secondary school students’ teacher support trajectories and their association with the development of study-related burnout and study engagement are explored.

Previous research has suggested that gender may influence students’ experience of teacher support. Girls tend to perceive higher levels of teacher support than boys (Li and Lerner, 2011; Lam et al., 2012; Liu et al., 2016; Wentzel et al., 2017), particularly in primary school (Liu et al., 2016). For example, Li and Lerner (2011) found that girls generally have more stable teacher support experiences. However, there is conflicting evidence on this topic. We recently showed that while girls perceived higher levels of support in the fourth grade, the levels decreased faster among girls than boys from the fourth to the sixth grade (Rautanen et al., 2022). Some studies have found no effect of gender on perceived teacher support (Wang and Eccles, 2012; Hughes and Cao, 2018; Özdemir and Özdemir, 2020). To better understand this variation, this study explores whether gender affects students’ perceived teacher support trajectories.

### 1.3. Aims of the study

The aim of this study is to explore whether the developmental trajectories of perceived teacher support vary among students, and are associated with experienced study engagement and study-related burnout (i.e., study-related exhaustion, cynicism and inadequacy). Also differences in teacher support trajectories between primary and lower-secondary school students and between boys and girls are explored. For this purpose, we identified distinct subgroups (i.e., classes) of primary (from 4th to 6th grade) and lower secondary school (from 7th to 9th grade) students and followed them for three years. The following hypotheses were tested:

*H1*: Considering that students’ teacher support experiences have been found to vary between students (Bokhorst et al., 2010; Bosman et al., 2018; Weyns et al., 2018; Özdemir and Özdemir, 2020), we expect to find different teacher support trajectories among primary and lower-secondary school students.

*H2*: Since teacher support has previously been shown to be related to students’ study well-being (Wang and Eccles, 2012; Quin, 2017; Ulmanen et al., 2022a), we presume that the teacher support trajectory will predict students’ experienced study engagement and study-related burnout. Students with higher levels of teacher support will experience more study engagement and less study-related burnout than students with a lower level of teacher support in both grade groups.

*H3*: Since older students have been found to report lower teacher support, we expect that lower-secondary school students are more likely represent trajectories with lower support than primary school students (Malecki and Demaray, 2002; Bokhorst et al., 2010; Ulmanen et al., 2016a, 2022b; Özdemir and Özdemir, 2020).

*H4*: Due to girls’ better school adjustment (Lam et al., 2012; Wang and Eccles, 2012; Liu et al., 2016), we expect that girls will score higher than boys on teacher support at the beginning of grades 4 and 7 and experience a slower rate of decline in support during primary and lower-secondary school.

## 2. Materials and methods

### 2.1. Study context

This study was conducted in Finnish comprehensive schools comprising both primary and lower-secondary school levels. In Finland, comprehensive school consists of 9 years of compulsory general schooling for all children aged 7–16. Primary schools comprise grades 1–6, and lower secondary schools comprise grades 7–9. In the primary school context, students have a class teacher who teaches most of the subjects, stays with the same student group for multiple years and thus provides the main source of teacher support for the students. Class teachers in Finland have Master’s degrees in educational sciences and are relatively independent in choosing the best pedagogical methods for their students when implementing the curriculum. At the lower-secondary level, a subject teacher system is applied. Lower-secondary school students have multiple subject teachers who typically do not spend as much time with each student as the class teachers do. Subject teachers have Master’s degrees in the subject domains that they teach (e.g., mathematics, languages, music) and 60 credits of pedagogical studies in the teacher training program. Thus, the emphasis of subject teachers’ education is more on content knowledge and less on pedagogical understanding, compared with that of class teachers’ education.

### 2.2. Sampling strategy and participants

Two cohorts of students (*N* = 3,615), namely primary and lower-secondary school students, from Finnish comprehensive schools participated in the study. They were followed for three years: the primary school students from 4th to 6th grade (N^T1^ = 2,204, 50% girls, age 10; N^T2^ = 2067, 50% girls, age 11; N^T3^ = 2003, 51% girls, age 12) and the lower-secondary school students from 7th to 9th grade (N^T1^ = 1,411, 51% girls, age 13; N^T2^ = 1,294, 51% girls, age 14; N^T3^ = 1,240, 51% girls, age 15). The participants were from 237 different class groups in 68 different Finnish comprehensive schools. The schools in the sample represented a demographic variety of the schools in Finland; that is, they were situated throughout the country and varied in size and location (rural/urban and high SES/low SES). SES refers to the levels of income, employment and education in the area surrounding the school. School size varied from 50 students to over 1,000 students, and class size varied from five to 33 students.

The data were collected from the participants as a part of the larger national School Matters research project by utilizing clustered hierarchical sampling (Snijders and Bosker, 2012) during three consecutive academic semesters (autumn 2017, autumn 2018, and autumn 2019). The members of the research group introduced the students to the study, instructed them on how to fill out the questionnaire and collected the written questionnaires from the students. Before the study was conducting, parents gave informed consent for their children to participate in the study. The students were informed that participating in the research was voluntary, that it was not a school assignment and that their teachers and parents would not see any individual student’s answers.

In Finland, an ethics review is required when research involves intervention in the physical integrity of research participants, deviates from the principle of informed consent, involves participants under the age of 15 being studied without parental consent, exposes participants to exceptionally strong stimuli, risks causing long-term mental harm beyond that encountered in normal life or signifies a security risk to subjects (Finnish National Board on Research Integrity, 2019, p. 19). None of these conditions were encountered in this study.

### 2.3. Measures

Following scales were used: (1) teacher support (11 items) (Rautanen et al., 2020), (2) study-related burnout (7 items) (see Salmela-Aro et al., 2009) and (3) study engagement (9 items) (see Salmela-Aro and Upadyaya, 2012). The teacher support scale was used to assess emotional support (i.e., respect, empathy, and care) and problem-focused informational support from teachers that helps students to achieve learning goals (α = 0.95 at T1, T2, and T3) (see Rautanen et al., 2020). The scale has been validated in previous studies (Rautanen et al., 2020, 2022; Ulmanen et al., 2022a,b).

The study-related burnout and study engagement scales were used to measure students’ study well-being. The former comprised three subscales to assess student study-related exhaustion (α = 0.73 at T1; α = 0.75 at T2; α = 0.77 at T3; 3 items), cynicism (α = 0.74 at T1; α = 0.80 at T2; α = 0.81 at T3; 2 items) and sense of inadequacy (α = 0.66 in grade 7, α = 0.73 at T2, α = 0.77 at T3; 2 items) (Supplementary Table S1). The scale was adapted from the School Burnout Inventory (SBI) (Salmela-Aro et al., 2009). Consistent with previous studies of older students, our confirmatory factor analyses (CFAs) supported the use of a three-factor structure of study-related burnout instead of the general factor structure (Salmela-Aro et al., 2009).

The study engagement scale was used to assess students’ energy, dedication, and absorption in studying (α = 0.94 at T1, T2 and T3) and was adapted from the Schoolwork Engagement Inventory (EDA) (Salmela-Aro and Upadyaya, 2012; Supplementary Table S1). In terms of study engagement, our CFAs supported the use of a general factor structure instead of the three-factor structure. All scales were rated on a seven-point scale ranging from 1 (“completely disagree”) to 7 (‘completely agree’). The items for all scales are presented in Supplementary Appendix A.

### 2.4. Preliminary analyses

First, students who dropped out of the study after T1 and did not return to it at T2 or T3 (*n* = 334) were removed from the analysis (see the final number of the students in Supplementary Appendix B Table B2). They perceived a slightly lower level of study engagement compared with students who answered at least two stages of the study (Cohen’s *d* = 1.44, *t*-test *p* = 0.03, mean difference = −0.18). Typical reasons for students to drop out from the study included that they were absent from school on the day data were collected or they had changed to another school that was not included in this study. Secondly, missing data analyses were conducted for used variables. Little’s MCAR test showed that across time points, the missing data were not completely random (χ^2^ = 4989.82, DF = 4,137, *p* = 0.000). The amount of missing data in the variables (%) is presented in Supplementary Table S3. The full-information maximum likelihood estimator (MLR) with robust standard errors was used to account for the missing data in further analyses.

CFAs were conducted to examine the general factor structure of the scales used in the study and their measurement invariance across three-time waves. The parameters of the models were estimated using the MLR estimation method available in Mplus 8.00 (Muthén and Muthén, 1998–2017) in conjunction with the complex option that considered the nesting of students in schools and classes (Muthén and Satorra, 1995; Peugh, 2010). The model fit was evaluated using the following criteria for adequate or good fit: the comparative fit index (CFI) and the Tucker–Lewis index (TLI) above 0.90/0.95, the root mean square error of approximation (RMSEA) below 0.08 (Hu and Bentler, 1999; Hooper et al., 2008). In addition, the chi square test statistic was used with caution because of its sensitivity to a large sample size. The measurement invariance of the latent constructs over time was tested by assessing (1) configural invariance, (2) metric invariance, and (3) scalar invariance (Cheung and Rensvold, 2002; Chen, 2007). A ∆CFI and ∆TLI of 0.010 or less and a ∆RMSEA of 0.015 or less between two subsequent models supports the invariance hypothesis (Cheung and Rensvold, 2002; Chen, 2007). CFAs achieved acceptable fit for each scale at each age cohort after modifications of two added residual covariances at maximum. Moreover, the longitudinal CFAs supported scalar invariance for each scale. The results of CFA are presented in Supplementary Appendix Table B1. In addition, the descriptive results of the variables are presented in Supplementary Appendix Table B2.

### 2.5. Latent growth mixture modeling

The initial step prior to specifying latent classes was to specify a single growth trajectory of teacher support that could adequately approximate an entire population by using latent growth curve modeling (LGCM). LGCM was used to examine whether there was variance between students in their initial level of (intercept) and development (slope) of perceived teacher support (Jung and Wickrama, 2008). We estimated linear growth curve models for teacher support and considered their fit with the data. Based on the fit indices considered (CFI, TLI, RMSEA and SRMR), the linear model for a single growth trajectory of teacher support showed a good fit for the data (*χ*^2^(1) = 5.41, *p* = 0.02, RMSEA = 0.035, CFI = 0.999, TLI = 0.996, SRMR = 0.013). The statistically significant chi-square value was probably due to the large sample size. The results of the single growth trajectory are presented in Table 1. The means of the latent variables were statistically highly significant (intercepts and slopes). The negative mean of the linear development (slope) describes that perceived teacher support decreased after the beginning of the first measurement point (i.e., after the 4th and 7th grades). Also, the variances of the latent variables were statistically significant, indicating variation in the baseline and the linear development of teacher support.

**Table 1:** Variances and means of general growth curve models and growth mixture model trajectories among primary and lower-secondary school students.

	Mean		Variance		I with S
Trajectories	Intercept (S.E.)	Slope (S.E.)	Intercept (S.E.)	Slope (S.E.)	
General model	5.172 (0.037)*	−0.147 (0.016)*	1.228 (0.057)*	0.267 (0.028)*	−0.189*
High stable (71.6%)	5.567 (0.035)*	−0.087 (0.019)*	0.541 (0.050)*	0.083 (0.024)*	−0.045
Low stable (12.4%)	3.381 (0.110)*	−0.088 (0.054)	0.541 (0.050)*	0.083 (0.024)*	−0.045
Decreasing (10.9%)	5.553 (0.084)*	−1.221 (0.066)*	0.541 (0.050)*	0.083 (0.024)*	−0.045
Increasing (5.1%)	3.124 (0.216)*	1.158 (0.117)*	0.541 (0.050)*	0.083 (0.024)*	−0.045

*p < 0.000 level.

Latent growth mixture modeling (LGM) was used to fully capture information about inter-individual differences in intra-individual changes in perceived teacher support (Li et al., 2001). The goal was to classify individuals into distinct groups or categories based on individual response patterns so that individuals within a group were more similar than individuals between groups considering unobserved heterogeneity (different groups) within a larger population. To determine the most appropriate number of latent trajectory classes, we examined theoretical conformity and the substantive meaning of the classes and the statistical compatibility of the class solution with the data (Nylund et al., 2007). The models were specified by estimating the variances of the latent variables as being equal in different groups (Jung and Wickrama, 2008). Firstly, the subsequent models were compared with the bootstrapped likelihood ratio test (BLRT). A statistically significant test result (*p* < 0.05) would indicate that a model with k classes fitted the data better than a model with one latent class fewer (i.e., k-1 classes) (Nylund et al., 2007). Secondly, Consitant Akaïke (CAIC), Bayesian (BIC) and sample size–adjusted BIC (ABIC) information criteria were employed to examine the goodness of fit of the model with the data (Bauer and Curran, 2003; Nylund et al., 2007). Lower values in these fit indices would indicate a better model fit. However, due to the large data size, the information criteria or the likelihood ratio test might not clearly support the choice of a particular class solution. The entropy values assess the accuracy with which models classify individuals into their most likely class, ranging from 0 to 1, with higher scores representing better a distinction between the latent profiles (Nylund et al., 2007).

We examined solutions including 1–6 latent classes for teacher support. The results of the information criteria and statistical significance for the different profile solutions are presented in Table 2. The information criteria decreased continuously without reaching minimum value. Similarly, the BLRT test was shown to be statistically significant until at least the sixth profile solution. The entropy values and the latent class probabilities were at a good level (above 0.7) until at least the sixth profile solution. Based on the theoretical and substantive examinations, four class models were chosen for further examination. Unlike the profile solutions from the first to the fourth, the fifth and sixth profile solutions contained the profiles with a small representativeness (2% or less). Moreover, adding a fifth class to the model did not result theoretically in more information about the different trajectories but divided the greatest class into the two smaller classes.

**Table 2:** Results from the latent trajectory analysis model estimated for teacher support.

k	LL	#fp	Scaling	CAIC	BIC	ABIC	Entropy	Size of trajectories (posterior prob.)	*P*_BLRT_
1	−15449.52	8	1.1993	30972.58	30964.58	30939.16	–		–
2	−15292.65	11	1.2688	30686.41	30675.41	30640.46	0.72	2962/652	0.000
3	−15205.88	14	1.2206	30540.45	30526.45	30481.97	0.74	2083/1278/252	0.000
4	−15153.84	17	1.2091	30463.94	30446.94	30392.93	0.74	2587/446/394/187	0.000
5	−15116.94	20	1.3671	30417.74	30397.74	30334.19	0.72	1874/1102/297/258/84	0.000
6	−15073.65	23	1.2651	30358.72	30335.72	30262.64	0.72	1857/927/340/226/194/70	0.000

k, number of classes; LL, model LogLikelihood; #fp, number of free parameters; Scaling, scaling factor; CAIC, consistent AIC; BIC, Bayesian information criteria; ABIC, sample size–adjusted BIC; VLMR, Vuong–Lo–Mendell–Rubin likelihood ratio test, aLRT, Lo–Mendell–Rubin adjusted likelihood ratio test; BLRT, bootstrap likelihood ratio test.

After identifying the most suitable latent profile solution, a BCH comparison was conducted to examine the mean differences of the outcome variables across the latent class trajectories. The BCH method is the most recommended method for examining relationships between latent class trajectories and continuous distal outcomes (in this study, engagement, and study-related burnout) (Asparouhov and Muthén, 2015). In addition, the R3STEP procedure, which performs a multinomial logistic regression describing the assumed effect of gender and grade on the likelihood of membership of each of the latent trajectories compared with other profiles, was used (Asparouhov and Muthén, 2015).

## 3. Results

**Figure 1:**
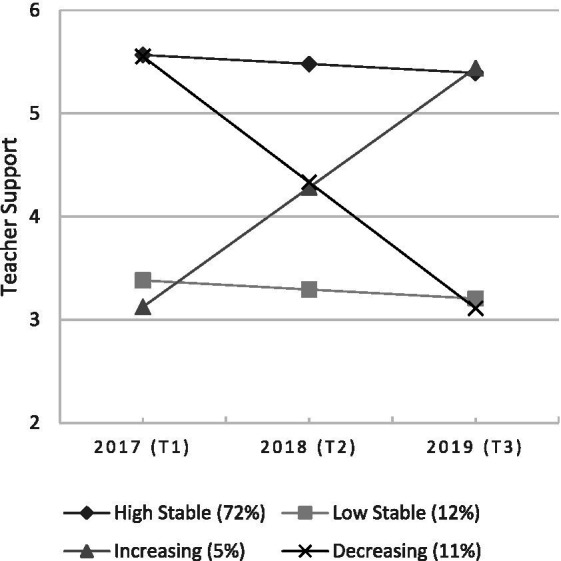
Three years’ latent class trajectories for students’ perceived teacher support.

Based on the LGM analysis, we identified four latent trajectories for teacher support, confirming the first hypothesis H1. The trajectories were labeled as *high stable, low stable, increasing* and *decreasing* (see Tables 1, 3 and Figure 1). Students representing the *high stable* trajectory reported teacher support above an average level throughout the measurement period, even if the perceived support decreased slightly in three years. Most of the students (72%) belonged to this stable class. The *low stable* trajectory represents students who reported teacher support significantly below an average level throughout the measurement period. Twelve percent of students represented this trajectory. The *increasing* trajectory represents students who reported teacher support approximately as low as students representing the *low stable* trajectory at the first measurement point, but their perceived support increased statistically significantly throughout the measurement period, being as high as or higher than students in the *high stable* trajectory had at the third measurement point. Five percent of students represented this class. Further, the *decreasing* trajectory represents students who reported teacher support as high as students representing the *high stable* trajectory at the first measurement point, but their perceived support decreased throughout the measurement period, being as low as students in the *low stable* trajectory had at the third measurement point. Eleven percent of students represented the *decreasing* latent trajectory.

**Table 3:** Differences in mean values between latent class trajectories of teacher support.

	High stable	Low stable	Increasing	Decreasing
Variable	Mean	Mean	Mean	Mean
Teacher supp T1	5.567	3.381	3.124	5.553
Teacher supp T2	5.480	3.293	4.282	4.332
Teacher supp T3	5.393	3.205	5.440	3.111

	High stable		Low stable		Increasing		Decreasing	
	Mean	S.E.	Mean	S.E.	Mean	S.E.	Mean	S.E.
Engagement T1	4.64	0.03	2.29^b^	0.09	2.65^b^	0.15	4.39	0.11
Engagement T2	4.43	0.03	2.26	0.09	3.50^a^	0.17	3.22^a^	0.11
Engagement T3	4.14^a^	0.00	2.07	0.10	4.29^a^	0.17	2.51	0.10
Exhaustion T1	2.80	0.03	4.32^a^	0.12	4.20^a^	0.18	3.38	0.12
Exhaustion T2	2.92	0.04	4.41	0.12	3.77^a^	0.20	3.66^a^	0.13
Exhaustion T3	3.16^b^	0.04	4.36^a^	0.12	3.29^b^	0.19	4.33^a^	0.13
Cynicism T1	2.05	0.03	3.94^a^	0.13	3.82^a^	0.20	2.40	0.12
Cynicism T2	2.13	0.04	4.17	0.14	3.11^a^	0.21	2.91^a^	0.13
Cynicism T3	2.16	0.04	4.12^a^	0.15	2.54	0.19	3.79^a^	0.15
Inadequacy T1	2.80	0.03	4.19^a^	0.11	4.36^a^	0.20	3.37	0.13
Inadequacy T2	2.78	0.04	4.58	0.13	3.52^a^	0.18	3.67^a^	0.13
Inadequacy T3	2.87^b^	0.04	4.57^a^	0.13	3.17^b^	0.21	4.49^a^	0.13

S.E., standard error. Means within a row sharing the same letter are not significantly different at the *p* < 0.05 level.

### 3.1. Study engagement and study-related burnout in different social support trajectories

The latent trajectory classes differed from each other in terms of study engagement and study-related burnout (Table 3). As expected in H2, higher levels of perceived school-related emotional and informational support from teachers were associated with higher levels of study engagement and lower levels of study-related burnout, while the lack of support raised the risk of study-related burnout and lack of study engagement. Moreover, the diminishing teacher support was related to a decline in study engagement and increase in study-related burnout. Perceptions of an increase in teacher support were related to an increase in study engagement and a decline in study related burnout.

Interestingly, students presenting *high stable* and *decreasing* trajectories perceived teacher support at a similar level at T1. However, students presenting the *decreasing* trajectory reported statistically significantly higher study-related exhaustion, inadequacy, and cynicism than students in the *high stable* trajectory at T1. Moreover, they reported statistically significantly lower study engagement than students in the *high stable* trajectory. It appears that perceiving higher levels of study burnout and lower study engagement predicts the risk for decreasing teacher support over time even if the beginning level of teacher support is high. This indicates that perceiving increased study-related exhaustion, inadequacy and cynicism and decreased study engagement raised students’ risk of isolating from teacher support as time progressed.

Moreover, students presenting the *low stable* and *increasing* trajectories perceived teacher support at a similar level at T1. However, students presenting the *increasing* trajectory reported slightly higher study engagement and slightly lower study-related exhaustion and cynicism than students in the *low stable* trajectory at T1. Although the difference was not statistically significant, it may indicate that perceiving higher study engagement predicts a positive change in perceived teacher support when the beginning level of teacher support is low in the primary school context. On the other hand, a lack of engagement is related to teacher support staying at a low level over time.

### 3.2. The roles of grade and gender

Results showed grade differences in students’ class membership (Table 4). In line with H3, lower-secondary school students, compared with primary school students, were more likely to belong to the *low stable, increasing* or *decreasing* trajectories than to the *high stable* trajectory. In addition, they were more likely to belong to the *low stable* trajectory than to the *decreasing* or *increasing* trajectory. In other words, they were more likely to represent trajectories with lower support than were primary school students.

**Table 4:** Results from multinomial logic regression for the effects of gender and grade on profile membership.

Variable	Low stable vs. high stable		Increasing vs. high stable		Decreasing vs. high stable	
	Coef. (*SE*)	*OR*	Coef. (*SE*)	*OR*	Coef. (*SE*)	*OR*
Gender	0.163 (0.157)	0.138	0.725 (0.253)**	0.661	−0.322 (0.168)*	−0.350
Grade	1.229 (0.163)***	1.202	0.499 (0.239)*	0.392	0.408 (0.167)*	380

	Increasing vs. low stable		Low stable vs. decreasing		Increasing vs. decreasing	
	Coef. (*SE*)	*OR*	Coef. (*SE*)	*OR*	Coef. (*SE*)	*OR*
Gender	0.561 (0.308)	0.466	0.485 (0.222)*	0.436	1.047 (0.284)***	0.966
Grade	−0.731 (0.298)*	−0.820	0.822 (0.226)***	0.771	0.091 (0.270)	0.018

***p = 0.000; **p < 0.01; *p < 0.05. SE, standard error of the coefficient; OR, odds ratio. Gender was coded 1 for girls and 2 for boys. Grade was coded 1 for primary and 2 for lower-secondary school students. The coefficients and OR reflect the effects of the predictors on the likelihood of membership into the first listed profile relative to the second listed profile.

Partly in line with H4, girls were more likely than boys to belong to the *high stable* or *decreasing* trajectories than to the *increasing* trajectory. In addition, girls were more likely to belong to the *decreasing* trajectory than to the *high stable* trajectory. In other words, girls perceived the initial level of teacher support to be higher than did boys. However, in contrast to H4, boys’ perceived teacher support was more likely to improve, while girls’ perceived teacher support to weaken.

## 4. Discussion

### 4.1. The findings considering previous research

The aim of this study was to explore individual differences between students in perceived teacher support trajectories and whether the trajectories were associated with experienced study well-being. For capturing the individual variation in students’ teacher support trajectories, latent growth mixture modeling was utilized. As we expected, several clearly different teacher support trajectories were found to reveal variation in perceived teacher support between students (Bosman et al., 2018; Weyns et al., 2018; Özdemir and Özdemir, 2020). In general, the results provide quite a positive view of the development of teacher support during the primary and lower-secondary school years. Seventy-two percent of students reported their teacher support to be high, although this slightly decreased throughout the study period. 5% of students reported a significant positive change. However, the results indicated a notable polarization between students, as almost a fifth of them reported constantly low support or a strong negative change (low stable and decreasing trajectories). Our results further suggested that the changes in teacher support are strongly related to a change in study engagement and study-related burnout (Wang and Eccles, 2012; Quin, 2017; Ulmanen et al., 2022a,b). Specifically, the results showed that higher levels of teacher support were associated with higher levels of study engagement and lower levels of study burnout symptoms, while lower levels of teacher support were associated with lower levels of study engagement and higher levels of study burnout symptoms.

The results confirm previous findings about the stability of teacher support experiences (Split et al., 2012; Bosman et al., 2018; Özdemir and Özdemir, 2020). Most of the students (84%) held trajectories whereby social support was perceived to be a quite stable, either at a low or a high level. Students who had positive experiences of teacher support in the past were more likely to perceive future teacher support to be high (72%). Similarly, students who experienced reduced teacher support were more likely to perceive it to be low in the future as well (12%). It is notable that while changes in perceived teacher support occurred, they were more likely to be negative than positive. This is, only 5% of students experienced an increase in perceived teacher support over time (i.e., the increasing trajectory), compared with 11% experiencing a decline (i.e., the decreasing trajectory). The result is in line with previous research showing that positive changes in teacher support can be more difficult to achieve (Jerome et al., 2009) since it often requires change in both teacher and student behavior and the way in which they perceive their relationship. Hence, mere change in teacher support behavior does not automatically result changes in student perceptions on teacher support. It is also possible that students perceiving a lack of teacher support face multiple diverse problems, including academic difficulties, social and emotional challenges or family issues (e.g., Ulmanen et al., 2022a). To address these issues effectively may require the expertise of a multi-professional team, since it may be difficult for an individual teacher to bring about positive change in all these areas.

The results suggest that decreased study well-being experience may trigger negative change in perceived teacher support (O'Connor, 2010; Ouweneel et al., 2011; Nurmi and Kiuru, 2015; Yang et al., 2021; Rautanen et al., 2022). Increased study-related burnout symptoms and decreased study engagement were associated with a decline in perceived teacher support, while higher levels of study engagement and low levels of study burnout symptoms were associated with a continuum of positive teacher support experience. It may be that both teacher and student characteristics contribute to how each relates and interacts (Saft and Pianta, 2001; Koles et al., 2009). It may be easier for teachers to provide appropriate support to students who are enthusiastic and feel that their schoolwork is important. In turn, engaged students may be more responsive and eager for support and ask for help with studies, compared with their less engaged peers. In turn, students with increased anxiety, cynicism and sense of inadequacy may not have the energy to engage with their teachers or seek help when needed. They are at risk of falling into a negative spiral, where negative study-related emotions and attitudes diminish the students’ means to seek and provide support (e.g., Hughes and Chen, 2011).

The results of grade differences show that primary school students were more likely to hold stable and high levels of teacher support, compared with lower-secondary school students. Specifically, lower-secondary school students were more likely to belong to the low stable, increasing, or decreasing trajectories than to the high stable trajectory. Additionally, they had a higher probability of being in the low stable trajectory than to the decreasing or increasing trajectory. Intriguingly, no statistically significant grade differences emerged between the decreasing and increasing trajectories meaning that both positive and negative changes are equally possible in both school contexts. The findings regarding the lower level of perceived teacher support in upper grade levels align with earlier research, which indicates a decline in perceived teacher support as students’ progress through their school years (Malecki and Demaray, 2002; Bokhorst et al., 2010; Ulmanen et al., 2016a, 2022b; Özdemir and Özdemir, 2020). However, the finding that both the decreasing and increasing trajectories are equally likely in both contexts is new. This suggests that while the experience of support is more prone to be at a lower level in the lower-secondary school setting, students in higher grades are not at a significantly heightened risk of transitioning to a diminishing support trajectory based on this study. Previous studies have often posited that the class teacher system may offer more changes for teachers to engage with and provide individualized support to their students in comparison to the subject teacher system (Jindal-Snape and Miller, 2008; Jindal-Snape et al., 2021). This study partially confirms these assumptions. However, it is a worth noting that the grade differences do not explain the significant changes in perceived teacher support. The strength of the class teacher system lies in the potential for class teachers to forge enduring relationships with their students, better attuning themselves to individual needs and concerns over time. Conversely, the subject teacher system’s advantage may lie in exposing individual students to multiple teachers throughout the school day. Consequently, any potential conflict or perceived pedagogical shortcomings with one teacher do not necessarily cast a shadow over a student’s entire school day or academic journey. Further research is needed to explore these potential factors in more detail.

Finally, some differences between boys and girls were found. In contrast to the hypothesis (Li and Lerner, 2011), the results showed that compared with boys, girls were statistically significantly more likely to belong to the *decreasing* trajectory than to the *increasing* trajectory. In addition, girls more probably belonged to the *decreasing* trajectory than to the *high stable* trajectory. Knowing that teacher support and study well-being experiences are strongly associated, the results are partly in line with previous studies of girls’ stronger tendency to experience study-related burnout, compared with boys (Kiuru et al., 2008; Salmela-Aro et al., 2008; Salmela-Aro and Tynkkynen, 2012). However, the results showing that increased study burnout symptoms trigger negative changes in teacher support, especially among girls, is new (see also Ulmanen et al., 2022a). It might be that girls rely on teachers more than boys to challenge school-related issues (see Ewing and Taylor, 2009). Thus, if support is perceived to be inappropriate or lacking, it may have more detrimental effects both on the development of study well-being and on teacher support (see McCormick and O'Connor, 2015). When interpreting the results, the small size of the increasing and decreasing trajectories should be considered.

### 4.2. Study limitations and future research

The study has several limitations. It is based on students’ self-reported measures, relying on the accuracy and honesty of the individual completing the measure. Using multiple informants, such as teachers and peers, and multiple methodologies, such as observations and interviews, could provide a more comprehensive perspective of social support and well-being. This is because different sources and methods may yield different information and provide a more holistic view of the individual’s experiences. It must also be considered that the scales for teacher support were only tested among Finnish students (Rautanen et al., 2020, 2022; Ulmanen et al., 2022a,b). It is important to test their validity in other countries and school systems as well. Additionally, the study’s generalizability is confined to Finnish primary and lower-secondary school students, potentially restricting its applicability to broader contexts. Future research should explore the relationships between teacher support trajectories and student well-being across various cultural backgrounds.

Moreover, this study focused only on the role of grade and gender in predicting individual variability in teacher support trajectories. While these factors may be important to consider, there may be other factors that also play a role in students’ experiences of teacher support. To achieve a more comprehensive and holistic perspective concerning individual-level variability, more predictors such as students’ individual characteristics would be important to consider in future research (e.g., Chen et al., 2018). It would be important to explore, for example, whether students’ social skills (Ciarrochi et al., 2002; Elias and Haynes, 2008) or a change in their family or peer relations influences students’ teacher support experiences (see Ulmanen et al., 2022a,b). Since sharing support is a reciprocal process, teachers’ personal characteristics should also be considered (e.g., Jennings and Greenberg, 2009; Nurmi and Kiuru, 2015). It would be relevant, for example, to study whether teachers’ professional agency or work-related burnout (see Tikkanen et al., 2021) relates to students’ teacher support trajectories. In addition, there have been indicative findings that student characteristics and behaviors evoke various responses from teachers and have a differing impact on teacher–student relationships (Houts et al., 2010; Nurmi, 2012). To sum up, further research is needed to identify factors that may predispose negative trajectories as well as factors triggering positive changes in students’ perceptions of teacher support and overall well-being.

### 4.3. Practical implications and conclusion

This study highlights the importance of understanding and addressing individual differences in the development of students’ perceived teacher support (e.g., Özdemir and Özdemir, 2020). The results revealed that students’ teacher support and study well-being experience are rather polarized, instead of being quite consistent experiences among students. To provide a consistent and supportive environment for all students, it is important for teachers to be aware of the individual differences in the way their support is perceived by students. For that, practices that would make it possible to check periodically how students gage their perceptions of teacher support would be helpful for teachers. Regular reciprocal communication with students and their families could also help to identify any areas of concern and to address them in a timely manner.

Moreover, the study revealed important findings about the reciprocity of perceived teacher support and study well-being (e.g., Rautanen et al., 2022). The results indicated that perceiving study burnout symptoms and decreased study engagement appear to be risk factors that may predict the beginning of a negative trajectory in teacher support. It is important for teachers and school staff to be aware of this dynamic and to provide sufficient support and resources to all students, regardless of their level of study well-being. To prevent a negative developmental trajectory in student’s social support and well-being in studies, special attention should be directed toward students who show study-related burnout symptoms, particularly a state of strain and chronic fatigue as well as a diminished sense of competence in terms of studying at school. This may involve providing individualized support to students who may be struggling as well as being open and responsive to the needs and concerns of all students.

Finally, the study highlights the importance of teacher support for students’ study well-being at all school levels (e.g., Ulmanen et al., 2022a). Special attention should be paid to improving the conditions in lower-secondary schools so that teacher support can reach all students. For example, subject teacher education could be developed in a way that provides better conditions for teachers to enhance pedagogy that considers students’ individual needs. As it stands, subject teacher education places a greater emphasis on the teacher’s subject-specific skills compared to pedagogical education in class teacher education, and this emphasis could potentially impact the level of social support perceived by students from their teachers. Moreover, improving the conditions and providing teachers with the resources and support they need to provide high-quality support to their students is important to have an immediate as well as lasting impact on students’ well-being and academic success.

## Data availability statement

The datasets presented in this article are not readily available due to ethical restrictions. Due to participant’s individual consent for using data only for scientific purposes within the research group, the gathered survey data cannot be shared publicly and supporting data are not available. Requests to access the datasets should be directed to the corresponding author: sanna.ulmanen@tuni.fi.

## Ethics statement

Ethical approval was not required for the study involving human samples in accordance with the local legislation and institutional requirements. Written informed consent for participation in this study was provided by the participants’ legal guardians/next of kin.

## Author contributions

SU had the main role in writing the original draft and in conducting the analyses. PR, KP, JP, and TS contributed to writing the original draft and editing the manuscript. KP, JP, and TS have contributed to funding acquisition and project administration. All authors contributed to the article and approved the submitted version.

## Funding

This research was supported by the Ministry of Education and Culture [grant number 6600567] and the Strategic Research Council (SRC), the Academy of Finland (grant number 1352509). SU was supported by a postdoctoral fellowship from the Finnish Cultural Foundation (grant number 00211124).

## Conflict of interest

The authors declare that the research was conducted in the absence of any commercial or financial relationships that could be construed as a potential conflict of interest.

## Publisher’s note

All claims expressed in this article are solely those of the authors and do not necessarily represent those of their affiliated organizations, or those of the publisher, the editors and the reviewers. Any product that may be evaluated in this article, or claim that may be made by its manufacturer, is not guaranteed or endorsed by the publisher.
