# Supplementary material for: How do teacher support trajectories influence primary and lower-secondary school students’ study well-being
Source: Front Psychol. 2023 Aug 24;14:1142469. doi: 10.3389/fpsyg.2023.1142469 (PMC10484616; doi:10.3389/fpsyg.2023.1142469)
Supplement: Supplementary file 1 [file Data_Sheet_1.docx]

Supplementary Material

**1 Appendix A**

*Items for the Scales of Teacher Support, Study-Related Burnout, and Study Engagement*

| Teacher support (11 items) |
| --- |
| 1. My teachers give me encouragement and support. 2. Problems are addressed in a constructive manner at my school. 3. I am treated with respect. 4. I often receive constructive feedback from teachers. 5. I am treated equally. 6. I can openly discuss problems related to my studies with teachers. 7. I feel that my teachers appreciate the work I have done for my studies. 8. The teachers are interested in my opinions. 9. I feel that my teachers care about me. 10. I often receive encouraging feedback from my teachers. 11. The teachers listen to the students at my school. |
| Study-related burnout (7 items) |
| 1. I feel drowned by my school work. 2. I feel like my studies are no longer important 3. I feel inadequate in relation to my studies. 4. I often sleep poorly due to issues with my school work. 5. Going to school feels like a waste of time to me 6. I spend a lot of time worrying about my studies outside of school time. 7. I often feel that I am failing in my studies |
| - Study engagement (9 items) |
| 1. When I study, I feel like I am bursting with energy. 2. I find my studies to be full of meaning and purpose. 3. Time flies when I’m studying. 4. When studying, I feel strong and vigorous. 5. I am enthusiastic about my studies. 6. When I am studying, I forget everything else around me. 7. My studies inspire me. 8. When I get up in the morning, I feel like going to class. 9. I can get carried away by my studies. |

Note. The items have been translated from Finnish. The item scale: 1 (completely disagree) to 7 (completely agree).

**2 Appendix B**

**Table B1**

*Goodness-of-Fit Statistics of the Longitudinal Confirmatory Factor Analytic Models*

| Model | χ²(*df)* | CFI | TLI | SRMR | RMSEA | 90% CI | Δχ2 (df) | ΔCFI | ΔTLI | ΔSRMR | ΔRMSEA |
| --- | --- | --- | --- | --- | --- | --- | --- | --- | --- | --- | --- |
| **Teacher support** | | | |  |  |  |  |  |  |  |  |
| T1 | 1050.320*(44) | .943 | .929 | .036 | .077 | .073-.081 |  |  |  |  |  |
| T2 | 699.117* (43) | .965 | .955 | .024 | .068 | .064-.073 |  |  |  |  |  |
| T3 | 814.087* (43) | .962 | .951 | .026 | .075 | .070-.079 |  |  |  |  |  |
| M1 | 3171.95*(456) | .958 | .952 | .028 | .042 | .040-.043 | - | - | - | - | - |
| M2 | 3226.82*(476) | .958 | .953 | .029 | .041 | .040-.042 | 31.76(20) | .000 | .001 | .001 | -.001 |
| M3 | 3469.28*(496) | .954 | .951 | .031 | .042 | .041-.043 | 268.00*(20) | -.004 | -.002 | .002 | .001 |
| **Study Burnout (three-factor model)** | | | | | | | | | | | |
| T1 | 239.868* (11) | .959 | .921 | .036 | .074 | .066-.082 |  |  |  |  |  |
| T2 | 271.301* (11) | .957 | .917 | .045 | .085 | .077-.094 |  |  |  |  |  |
| T3 | 222.593* (9) | .969 | .928 | .035 | .086 | .077-.096 |  |  |  |  |  |
| M1 | 964.89*(132) | .964 | .944 | .040 | .043 | .040-.046 | - | - | - | - | - |
| M2 | 987.367*(140) | .964 | .946 | .041 | .042 | .040-.045 | 13.65(8) | .000 | .002 | -.001 | -.001 |
| M3 | 1025.39*(148) | .963 | .947 | .041 | .042 | .039-.044 | 35.49*(8) | -.001 | .001 | .000 | .000 |
| **Study engagement** | | | | | | |  |  |  |  |  |
| T1 | 344.145*(27) | .979 | .972 | .021 | .056 | .050-.061 |  |  |  |  |  |
| T2 | 455.400*(27) | .973 | .964 | .022 | .070 | .064-.075 |  |  |  |  |  |
| T3 | 416.322*(27) | .974 | .966 | .021 | .067 | .061-.073 |  |  |  |  |  |
| M1 | 1630.633*(294) | .975 | .971 | .021 | .036 | .035-.038 | - | - | - | - | - |
| M2 | 1735.07*(310) | .974 | .970 | .024 | .037 | .035-.038 | 110.18*(16) | -.001 | -.001 | .003 | .001 |
| M3 | 1876.50*(326) | .971 | .969 | .026 | .037 | .036-.039 | 151.04*(16) | -.003 | -.001 | .002 | .000 |

*Note.* M1 = Configural model, M2 = Metric model, M3 = Scalar model; χ2 = chi-squared test of exact fit; *df* = degrees of freedom; CFI = Comparative Fit Index; TLI = Tucker–Lewis Index; RMSEA = root mean square error of approximation; 90% CI = 90% confidence interval of the RMSEA; Δχ2 = chi-square difference test, **p*<.01.

**Table B2**

*Observed Variables Correlations, their Means and Standard deviations*

| Variables |  | |  | | Correlations | | | | | | | | | | | | |
| --- | --- | --- | --- | --- | --- | --- | --- | --- | --- | --- | --- | --- | --- | --- | --- | --- | --- |
|  | *1* | *2* | | *3* | | *4* | *5* | *6* | *7* | *8* | *9* | *10* | *11* | *12* | *13* | *14* | *15* |
| *1* Teacher support T1 | - |  | |  | |  |  |  |  |  |  |  |  |  |  |  |  |
| *2* Teacher support T2 | .61 | - | |  | |  |  |  |  |  |  |  |  |  |  |  |  |
| *3* Teacher support T2 | .49 | .65 | | - | |  |  |  |  |  |  |  |  |  |  |  |  |
| 4 Engagement T1 | .62 | .45 | | .38 | | - |  |  |  |  |  |  |  |  |  |  |  |
| 5 Engagement T2 | .42 | .63 | | .47 | | .62 | - |  |  |  |  |  |  |  |  |  |  |
| 6 Engagement T3 | .31 | .40 | | .57 | | .51 | .65 | - |  |  |  |  |  |  |  |  |  |
| 7 Exhaustion T1 | -.35 | -.30 | | -.27 | | -.31 | -.27 | -.21 | - |  |  |  |  |  |  |  |  |
| 8 Exhaustion T2 | -.28 | -.37 | | -.30 | | -.26 | -.33 | -.23 | .49 | - |  |  |  |  |  |  |  |
| 9 Exhaustion T3 | -.20 | -.26 | | -.34 | | -.18 | -.24 | -.30 | .38 | .52 | - |  |  |  |  |  |  |
| *10* Cynicism T1 | -.44 | -.34 | | -.27 | | -.50 | -.39 | -.28 | .49 | .24 | .14 | - |  |  |  |  |  |
| *11* Cynicism T2 | -.34 | -.48 | | -.34 | | -.39 | -.56 | -.36 | .32 | .46 | .23 | .49 | - |  |  |  |  |
| *12* Cynicism T3 | -.28 | -.35 | | -.44 | | -.31 | -.41 | -.55 | .26 | .28 | .43 | .38 | .50 | - |  |  |  |
| *13* Inadequacy T1 | -.35 | -.28 | | -.26 | | -.33 | -.27 | -.20 | .66 | .37 | .27 | .53 | .33 | .28 | - |  |  |
| 14 Inadequacy T2 | -.32 | -.42 | | -.32 | | -.28 | -.37 | -.26 | .41 | .69 | .41 | .30 | .54 | .34 | .44 | - |  |
| 15 Inadequacy T3 | -.25 | -.31 | | -.41 | | -.22 | -.29 | -.36 | .35 | .43 | .71 | .21 | .29 | .52 | .36 | .49 |  |
| N | 3515 | 3283 | | 3222 | | 3496 | 3279 | 3221 | 3515 | 3285 | 3222 | 3512 | 3283 | 3219 | 3498 | 3272 | 3215 |
| Mean | 5.16 | 5.07 | | 4.89 | | 4.22 | 3.98 | 3.72 | 3.13 | 3.23 | 3.44 | 2.42 | 2.52 | 2.60 | 3.11 | 3.14 | 3.27 |
| SD | 1.27 | 1.36 | | 1.35 | | 1.44 | 1.45 | 1.42 | 1.55 | 1.57 | 1.60 | 1.55 | 1.62 | 1.60 | 1.58 | 1.62 | 1.69 |
| Missing data (%) | 2.8 | 2.3 | | 0.6 | | 3.3 | 2.4 | 0.7 | 2.8 | 2.3 | 0.6 | 2.8 | 2.3 | 0.7 | 3.2 | 2.6 | 0.9 |
| Scale | 1-7 | 1-7 | | 1-7 | | 1-7 | 1-7 | 1-7 | 1-7 | 1-7 | 1-7 | 1-7 | 1-7 | 1-7 | 1-7 | 1-7 | 1-7 |

*Note.* All correlations were statistically significant at the p=.000 level.
